# Bioactive Compounds and Stability of a Typical Italian Bakery Products “*Taralli*” Enriched with Fermented Olive Paste

**DOI:** 10.3390/molecules24183258

**Published:** 2019-09-06

**Authors:** Miriana Durante, Gianluca Bleve, Roberto Selvaggini, Gianluca Veneziani, Maurizio Servili, Giovanni Mita

**Affiliations:** 1 Institute of Sciences of Food Production-CNR, Via Monteroni, 73100 Lecce, Italy; 2 Department of Agricultural, Food and Environmental Sciences, University of Perugia, Via S. Costanzo, 06126 Perugia, Italy

**Keywords:** carotenoids, enriched foods, food by-products, hexanal, polyphenols, tocochromanols, triterpenic acids

## Abstract

Olive paste (OP) is a novel by-product of olive mill industry composed of water, olive pulp, and skin. Due to its richness in bioactive compounds, OP exploitation for human consumption has recently been proposed. Starter driven fermented OP is characterized by a well-balanced lipid profile, rich in mono and polyunsaturated fatty acids, and a very good oxidative stability due to the high concentration of fat-soluble antioxidants. These characteristics make OP particularly suitable as a functional ingredient for food/feed industry, as well as for the formulation of nutraceutical products. New types of *taralli* were produced by adding 20% of fermented OP from black olives (cv Cellina di Nardò and Leccino) to the dough. The levels of bioactive compounds (polyphenols, triterpenic acids, tocochromanols, and carotenoids), as well as the fatty acid profile, were monitored during 180 days of storage and compared with control *taralli* produced with the same flour without OP supplementation. *Taralli* enriched with fermented OP showed significantly higher levels of bioactive compounds than conventional ones. Furthermore, enriched *taralli* maintained a low amount of saturated fatty acids and high levels of polyphenols, triterpenic acids, tocochromanols, and carotenoids, compared to the initial value, up to about 90 days in the usual conditions of retailer shelves.

## 1. Introduction

Fruits and vegetables are known to be rich sources of several bioactive compounds, also known as nutraceuticals [1,2]. Agri-food by-products produced during handling and processing of fruits and vegetables, including residual pulp, peels, seeds, leaves, bracts, stems, roots, and bark, represent a major waste disposal problem for industry [3]. Nevertheless, food industry by-products can be good and low-cost sources of valuable bioactive compounds, such as polyphenols, carotenoids, tocochromanols, and phytosterols [4,5]. The bioactive compounds could be extracted, or some by-products could be directly used as ingredients for foods/feed supplementation. Various extraction techniques have been applied from a waste range of plant materials to obtain phytocomplexes rich in high-value components [6,7,8] useful for the industrial preparation of functional foods, dietary supplements, and/or cosmeceuticals. Furthermore, some constituents of the phytocomplex may act as an anticancer agent, anti-inflammatory, and antioxidant or have a role in cell signaling and gene expression regulation, maintaining our health [9]. The olive fruit is an excellent source of unsaturated fatty acids, as well as other nutritionally important health-promoting bioactive compounds [10]. Olive mill industry generates large amounts of different by-products. Among these, olive pomace, olive mill wastewaters, and the olive stones and seeds have attracted attention for their possible exploitation in several industrial sectors. The recently introduced multi-phase decanter technology for olive oil industrial extraction generates large quantities of a novel by-product (olive paste—OP) made up of the partially defatted wet drupe pulp containing very low or any traces of the kernel. Recently, Padalino et al. [11], Cecchi et al. [12], and Tufariello et al. [13] reported that OP is an excellent natural source of unsaturated fatty acids and biologically active substances, including polyphenols, triterpenic acids, tocochromanols, and carotenoids.

Nowadays, the increasing demand for high nutritive value and health-promoting foods encourages the food industry to develop research towards innovative products containing higher concentrations of nutrients and bioactives. Studies have demonstrated that cereal-based food products, such as pasta and bakery products, represent cheap and easy-to-use carriers for phytochemicals. The most recent enrichment of pasta includes a variety of non-traditional ingredients, such as lipophilic (tocochromanols, carotenoids) or hydrophilic/phenolic antioxidants from durum wheat bran by-products, lyophilized tomato containing high levels of lycopene [14], α-cyclodextrin chlatrated pumpkin oil rich in α-, β- carotene and α-tocopherol [15], OP powder rich in maslinic acid and polyphenols, such as tyrosol [11]. 

Fermentation approaches using microorganisms selected to grow in the presence of high levels of phenolic bioactive compounds have been extensively and successfully applied to olives and derived products [16,17,18]. In a previous study, the biotechnological aptitude, of yeast and lactic acid bacteria selected from different sources, was tested to transform OP in a new fermented product with quality and safety traits suitable to be used as a semi-finished ingredient for human food formulation. 

The yeast strain *Saccharomyces cerevisiae* KI-30-1 and the strain *Leuconostoc mesenteroides* BC-T3-35 were selected among other candidates as the best performing microorganisms for their metabolic activities able to consume sugars, to produce important organic acids and volatile compounds, and to substantially modify phenolic profiles, by improving nutritional traits of the final product. Indeed, fermented OPs obtained from black olives of the cultivar Cellina di Nardò (OPC) and Leccino (OPL) were shown to represent a good source of bioactive compounds [13]. These strains were used for the first time as starters for pilot-scale fermentations of OP following a sequential inoculation approach, by mimicking the already described process in table olives fermentation, since yeasts are fundamental to modify the product and to prepare the subsequent lactic acid bacteria (LAB) fermentation [13,17,18].

Fermented OP could be then exploited to enrich bakery products, such as *taralli,* enhancing their nutritional and health-promoting properties. *Taralli*, are a typical Italian bakery product produced in many Italian regions, mainly in Central and Southern Italy [19], very popular worldwide as a savory snack or bread substitute and characterized by a high friability and long shelf-life. Their formulation includes fats, which confer desirable texture, flavor, friability, and crispy consistency [20]. Fats added to the dough are usually vegetable oils from corn, palm, soybean, sunflower, and, more frequently, olive. Fats also influence the oxidative processes of food and then its shelf life. Caponio et al. [21,22] and Giarnetti et al. [23] reported that oil typology plays an important role in the formation and release of volatile aromas during *taralli* storage. The authors reported that volatile compounds increased during storage and that the content of individual volatile molecules was higher in *taralli* made with Extra-Virgin Olive Oil (EVOO) than refined oils.

The aim of this work was the formulation and characterization of innovative enriched *taralli* containing 20% of fermented OP to improve the nutritional and health value of the product. Furthermore, the content of bioactive compounds, the profiles of fatty acids, and the production of hexanal were monitored over 180 days storage at 25 °C in light conditions in enriched and conventional (control) *taralli*. 

## 2. Results and Discussion

### 2.1. Chemical Characterization of Control and Enriched Taralli

Table 1 shows the profile of fatty acids and the chemical composition of polyphenols, triterpenic acids, tocochromanols, and carotenoids in conventional and enriched *taralli* obtained adding 20% of the fermented OP to the dough. The amount of OP added to the dough was determined by preliminary trials aimed to achieve a significant increase in bioactive compounds without excessively affecting the rheological properties of the *taralli* compared with the control (CTRL) ones (data not shown). Indeed, the dough containing more than 20% fermented OP presented low specific volume, while the resulting *taralli* were harder and less leavened than CTRL.

Lipids play a key role in bakery products, especially biscuits, cookies, and *taralli*, as they affect the rheological behavior, sensory properties, nutritional value, and shelf life of the final product [21,24]. The fatty acid profiles of CTRL and enriched *taralli*, expressed as a relative percentage with respect to the total identified molecules, did not show statistically significant differences (*p* > 0.05). Both comprised mainly mono-unsaturated fatty acids (MUFA; 68%); saturated fatty acids (SFA; 18%), and poly-unsaturated fatty acids (PUFA; 14%). Oleic acid (C18:1 n-9) contributed about 66% of the total fatty acids identified, followed by palmitic (C16:0; 15%) and linoleic (C18:2n-6; 13%) acids. Although the fatty acid profile of CTRL and enriched *taralli* is influenced by the lipid composition of wheat flour and vegetable oil used as ingredients, it was almost identical to that of *taralli* made with olive oil, as also reported by Caponio et al. [22]. 

*Taralli* obtained adding fermented OP contained several beneficial compounds, including polyphenols (1377 µg/g dried weight (DW) in *taralli* OPC and 1016 µg/g DW in *taralli* OPL), triterpenic acids (80.26 µg/g DW in *taralli* OPC and 80.92 µg/g DW in *taralli* OPL), typically absent in conventional *taralli* [13], isoprenoids, such as tocochromanols (30.27 µg/g DW in *taralli* OPC and 22.22 µg/g DW in *taralli* OPL) and carotenoids (1.17 µg/g DW in *taralli* OPC and 1.03 µg/g DW in t*aralli* OPL). In conventional *taralli,* the amount of these isoprenoids were lower. 

Various agri-food by-products rich in polyphenols, including pomegranate, mango, pigeon pea, and apple peels, have been mixed to the dough to prepare bakery products with functional properties. Some researchers reported that the supplementation of polyphenols in bakery foods could decrease starch digestibility and reduce the postprandial glucose levels in the serum, and is thus considered as alternatives to pharmaceutical interventions for the treatment of type II diabetes [25,26,27,28]. In this work, the addition of fermented OP to durum wheat flour led to a significant enrichment of polyphenol compounds. 

It is worthwhile noting that although durum wheat is known to be a source of phenolics, most of them are in the form of insoluble phenolic acids bound by covalent cross-linkages to the cell wall polymers (75% of the total phenolic acids) and thus have poor bioavailability [29]. The soluble conjugated and free forms represent about 24% and 1%, respectively [29]. In this work, we focused the attention on the free polyphenol fraction. 

The supplementation with the OP strongly increased polyphenols concentrations in the OPC and OPL *taralli* compared with CTRL sample (Figure S1). This is probably related to the fact that polyphenols content in EVO added to the CTRL dough was low, and it was not detectable in the final product (Table S1). In OP enriched *taralli,* the main polyphenol compound found was hydroxytyrosol (66% and 77% of the total polyphenols identified in OPC and OPL *taralli*, respectively) followed by verbascoside. Hydroxytyrosol possesses many beneficial properties for human health, and it is currently used as a therapeutic agent [30]. Recently, Difonzo et al. [31] reported that olive leaf extract (OLE), rich in polyphenols (i.e., oleuropein, verbascoside, luteolin, rutin, hydroxytyrosol, and tyrosol), was also suitable to enhance the quality of bakery products. 

Triterpenic acids, from olive by-products, such as maslinic and oleanoic acids, are mainly concentrated in the skin of drupes [11,13,32,33]. These compounds have received great attention because of their functional properties and biological importance [34]. Recently, Sanchez Rodriguez et al. [35] reported that a daily supplementation during three weeks with 30 mL of an enriched virgin olive oil providing 4.7 mg/d of oleanoic acid (171 ppm) and 6 mg/d of maslinic acid (218 ppm) decreased DNA oxidation and plasma inflammatory biomarkers in healthy adults compared to the conventional oil with low levels of triterpenic acids. In our study, *taralli* enriched with fermented OP showed the presence of both maslinic and oleanoic acids (Table 1). 

The fat-soluble micronutrients, tocochromanols, occur in different forms (α-, β-, γ-, and δ-tocopherols and α-, β-, γ-, and δ-tocotrienols) and are known to possess many health benefits, including protection against cancer, cardiovascular, and age-related degenerative diseases [36,37]. The total tocochromanols content of OPC and OPL enriched *taralli* was about 2.1- and 1.6-times, respectively, higher than CTRL. The main tocochromanols were α-tocopherol (α-T) and β-tocotrienol (β-T3). It is known that α-T is the only form of tocopherols that is actively maintained in the human body [38]. In conventional *taralli*, α-T content was 10.31 μg/g DW similar to the amount reported by Leenhardt et al. [39] in bread obtained from *Triticum monococcum* (11.03 μg/g) and *durum* (9.46 μg/g). In enriched *taralli*, α-T content ranged from 15.63 μg/g DW (*taralli* OPL) to 21.26 μg/g DW (*taralli* OPC), these values are in agreement with the study of Pasias et al. [40] who reported that bakery products (i.e., breadstick) made from *Triticum dicoccum* wheat and other ingredients, such as vanilla, tomato, sesame, and/or vegetables, gave a high content of α-T that ranged from 18.2 μg/g to 88 μg/g. β-T3 content ranged from 6.59 μg/g DW (*taralli* OPL) to 9.01 μg/g DW (*taralli* OPC). 

In all samples, lutein was the main carotenoid, followed by β-carotene. *Taralli* enriched with fermented OP showed a small, but statistically significant, increase in total carotenoids levels with respect to CTRL *taralli*. In general, bioactive compounds profile in *taralli* was in agreement with the study of Padalino et al. [11] on spaghetti enriched with olive paste. These authors demonstrated that the innovative spaghetti had high bioactive compounds content. In particular, the results showed that levels of apigenin, luteolin, quercetin, maslinic, and oleanoic acids and α-T, α-, and β-carotene, observed in enriched spaghetti, were higher than in conventional spaghetti. 

### 2.2. Effect of Storage on Fatty Acids Profile and the Content of Hexanal, Polyphenols, Triterpenic Acids, Tocochromanols, and Carotenoids in Conventional and Enriched Taralli

The effect of light exposure, the most important factor influencing *taralli* shelf-life quality during supermarket storage, was evaluated by determining the fatty acids profile, the amount of hexanal, polyphenols, triterpenic acids, tocochromanols, and carotenoids at 30 day-intervals during storage for 180 days. 

Lipid oxidation is the main biochemical process responsible for the deterioration of bakery products and reduction of their shelf life. The oxidation reaction depends on the type of fats used and product composition [20,41]. Table 2 shows the changes (%) in SFA, MUFA, and PUFA in conventional and enriched *taralli* during storage. In OPC *taralli,* the SFA percentage did not vary significantly, while some variations were observed in CTRL (*p* = 0.044) and OPL *taralli* (*p* = 0.013) after 90 and 120 days storage, respectively, compared to the initial value. In all tested samples, the results showed no significant variation in MUFA percentage. The percentage of PUFA, instead, underwent a significant decrease after 60 days of storage in CTRL. In enriched *taralli,* the decrease in the percentage of PUFA was less marked, in particular, it significantly decreased after 120 (*p* = 0.001; OPC *taralli*) and 150 days (*p* = 0.029; OPL *taralli*) of storage. The PUFA/SFA ratio is one of the main parameters used to assess the nutritional quality of the lipid fraction of foods [42]. Nutritional guidelines recommend a PUFA/SFA ratio above 0.4 [43]. In all samples, PUFA/SFA ratios were significantly reduced during storage; however, the values observed, after 180 days of storage, in OPC (0.39) and OPL *taralli* (0.43) were higher than the CTRL *taralli* (0.25). This is in agreement with results reported by Padalino et al. [11] in spaghetti enriched with OP. Also, in that case, the PUFA/SFA ratio resulted higher in enriched spaghetti than in CTRL. The presence of antioxidants in fermented OP, such as polyphenols, tocochromanols, and carotenoids, could play an important role in oxidative protection to the PUFA in enriched *taralli*. Indeed, some of the natural antioxidants, such as α-T and β-carotene, have been already used to enhance the shelf life of bakery products [44]. Furthermore, recent studies have reported that the OLE, with a high content of phenolic compounds, can reduce the level of oxidation in bakery products, enhancing their quality and shelf-life [27,45].

**Figure 1:**
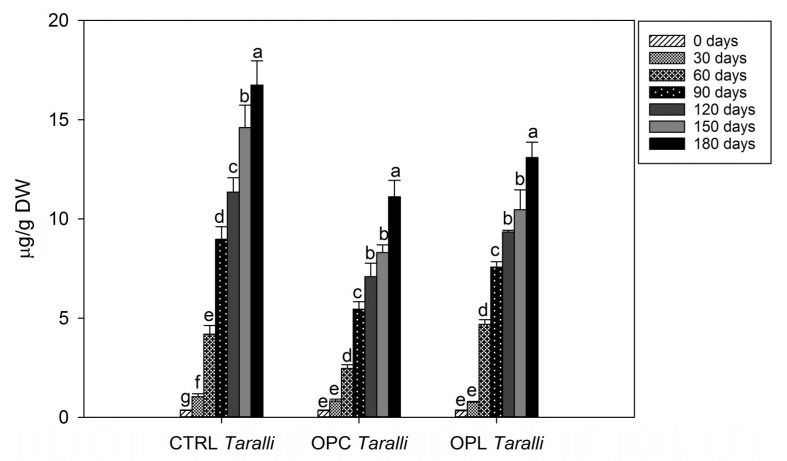
Time course of the amounts of hexanal during the storage of control (CTRL) and *taralli* enriched with fermented olive paste from Cellina di Nardò (OPC) or Leccino (OPL) olive cultivars at 25 °C, 500 lux, and in clear plastic bag for 180 days. Data are the mean ± standard deviation of three independent replicates (*n* = 3). Data were submitted to one-way analysis of variance (ANOVA), Tukey’s test was applied to compare the experimental times (0, 30, 60, 90, 120, 150, 180 days) within the same sample. Different letters mean significant difference at *p* < 0.05.

To evaluate the storage stability of *taralli* samples, we evaluated the increase of hexanal, the most abundant lipid oxidation volatile compound deriving from the oxidation of linoleic acid [46]. Hexanal has been already reported in several cereal-based foods, including pasta, bread, and biscuits. The lipid fraction of semolina, the main ingredient of bakery products, is very susceptible to lipoxygenase activity leading to hydroperoxide productions [47,48]. Hydroperoxides are highly unstable and are converted in volatile compounds, such as hexanal, responsible for rancid off-flavors [49]. Furthermore, hexanal is a representative marker of the oxidative rancidity as an alternative to traditional oxidation indicators (e.g., acidity or peroxide values). In this work, CTRL *taralli* exhibited a significative increase of hexanal within the first 30 days of storage, whereas, in enriched *taralli,* hexanal levels increased after 30 days of storage (Figure 1). Moreover, at the end of storage, in CTRL *taralli*, hexanal was observed at higher levels than enriched *taralli* (16.75, 11.11, 13.09 µg/g DW in CTRL, OPC, and OPL *taralli*, respectively). It could be considered that antioxidant compounds in fermented OP added to durum wheat flour were able to reduce the lipid oxidation in enriched *taralli* products. Similar results were reported by Difonzo et al. [31], who observed that OLE improved lipid stability in baked snacks compared to CTRL.

**Figure 2:**
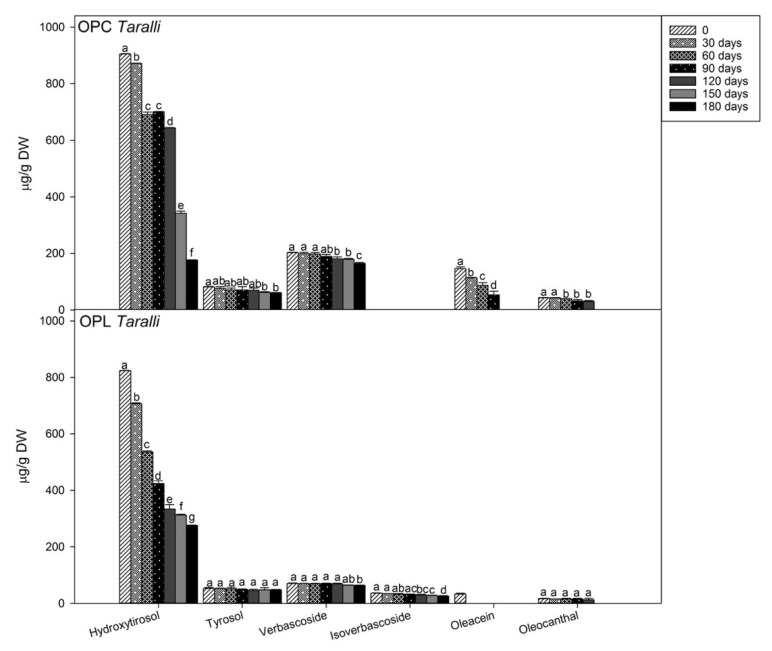
Time course of the amounts of polyphenols during the storage of CTRL and *taralli* enriched with fermented olive paste from Cellina di Nardò (OPC) or Leccino (OPL) olive cultivars at 25 °C, 500 lux, and in clear plastic bag for 180 days. Data are the mean ± standard deviation of three independent replicates (*n* = 3). Data were submitted to one-way analysis of variance (ANOVA), Tukey’s test was applied to compare the experimental times (0, 30, 60, 90, 120, 150, 180 days) within the same sample for each compound (hydroxytyrosol, tyrosol, verbascoside, isoverbascoside, oleacein, and oleocanthal). Different letters mean significant difference at *p* < 0.05.

Figure 2 shows the polyphenols profile in enriched *taralli* during storage. The content of total polyphenols progressively decreased during storage, leading to deterioration as a consequence of oxidative degradations probably due to light exposure. However, individual polyphenols showed a different decrease in trends over the storage period. In OPC and OPL *taralli*, the difference of hydroxytyrosol content after 30 days of storage was small (although statistically significant, *p* < 0.001), then a dramatic decrease by about 62% of the initial content occurred between 120 and 180 days of storage. The content of tyrosol, verbascoside, and isoverbascoside was, instead, more stable over time. Tyrosol level significantly decreased only by 23% to the initial value after 150 days of storage in OPC *taralli*, while it remained stable in OPL *taralli.* Esposto et al. [50] and Servili et al. [51] reported that tyrosol has lower antioxidant activity than hydroxytyrosol. Thus, hydroxytyrosol presented a higher degradation rate than tyrosol in *taralli* during storage in the presence of light. A small reduction of verbascoside levels (6–8%) after 120 (*p* = 0.002) and 180 (*p* = 0.017) days of storage was also observed in OPC and OPL *taralli*, respectively. Isoverbascoside content, not detectable in OPC *taralli*, was observed at low levels in OPL, and its content slowly decreased during storage to 26 µg/g DW. Also, oleacein level in OPL *taralli* gradually decreased during storage. Oleocanthal, present at the low but detectable amount, resulted undetectable after 120 days of storage both in OPC and OPL *taralli*.

**Figure 3:**
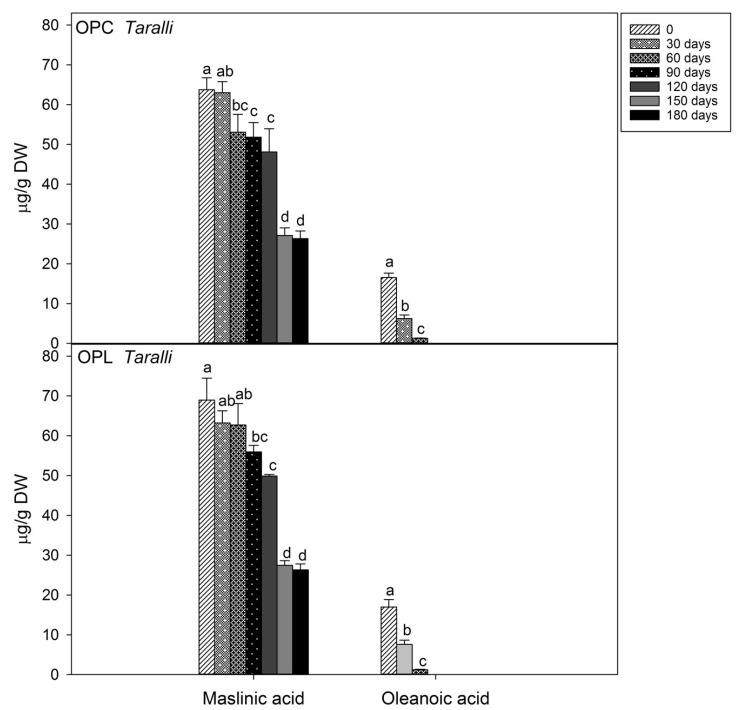
Time course of the amounts of triterpenic acids during the storage of CTRL and *taralli* enriched with fermented olive paste from Cellina di Nardò (OPC) or Leccino (OPL) olive cultivars at 25 °C, 500 lux, and in clear plastic bag for 180 days. Data are the mean ± standard deviation of three independent replicates (*n* = 3). Data were submitted to one-way analysis of variance (ANOVA), Tukey’s test was applied to compare the experimental times (0, 30, 60, 90, 120, 150, 180 days) within the same sample for each compound (maslinic and oleanoic acid). Different letters mean significant difference at *p* < 0.05.

In Figure 3, the effect of storage time on the content of triterpenic acids (maslinic and oleanoic acids) is reported. In *taralli* enriched with the fermented OP, maslinic acid content decreased slowly by 25 and 20% (OPL and OPC, respectively) after 120 days of storage. A dramatic decrease to less than 59%, in OPL, and 62%, in OPC, of the initial content occurred between 120 and 180 days of storage. In contrast, oleanoic acid rapidly decreased under the condition tested. After 60 days of storage, oleanoic acid level resulted reduced by approximately 93% in both samples. 

**Figure 4:**
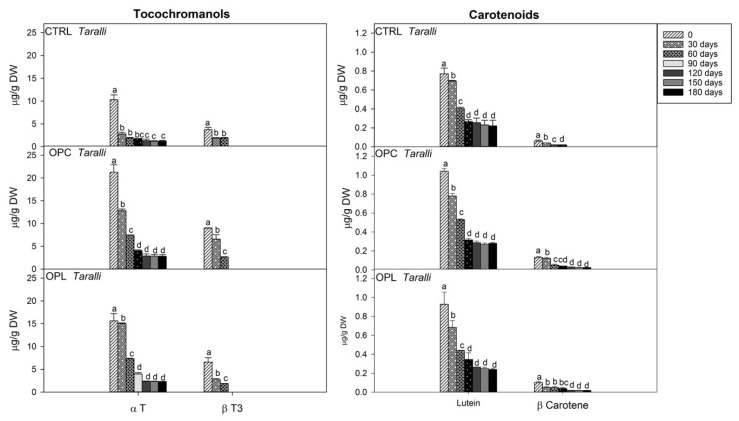
Time course of the amounts of tocochromanols and carotenoids during the storage of CTRL and *taralli* enriched with the fermented olive paste from Cellina di Nardò (OPC) or Leccino (OPL) olive cultivars at 25 °C, 500 lux, and in clear plastic bag for 180 days. Data are the mean ± standard deviation of three independent replicates (*n* = 3). Data were submitted to one-way analysis of variance (ANOVA), Tukey’s test was applied to compare the experimental times (0, 30, 60, 90, 120, 150, 180 days) within the same sample for each compound (α-T (α-tocopherol), β-T3 (β-tocotrienol), β-carotene, and lutein). Different letters mean significant difference at *p* < 0.05.

In Figure 4, the time course of the changes in the level of tocochromanols: α-T and β-T3 and carotenoids: β-carotene and lutein in CTRL and enriched *taralli* is reported. 

In CTRL *taralli*, after 30 days of storage, α-T concentration showed a rapid decrease by 72% of the initial value and its content was 1.24 µg/g DW after 180 days of storage. The level of α-T was reduced by 80% after 90 days of storage, then remained almost unchanged in OPC (2.81 µg/g DW) and OPL (2.32 µg/g DW) *taralli* up to 180 days. β-T3 content progressively decreased in all samples and was undetectable after 90 days of storage. β-T3 is more sensitive and susceptible to oxidation compared to α-T likely in relation to the presence of three, rather than two unsaturations [52]. A similar trend was observed by Durante et al. [6,8], who evaluated the stability of different tocochromanol forms in vegetable oils. 

The content of total carotenoids was reduced by more than 70% in all samples after 90 days of storage. In all samples, the amount of lutein progressively decreased over time, and its content was about 0.20 µg/g DW at the end of the storage period. After 90 days of storage, the β-carotene level was completely reduced in CTRL *taralli,* whereas, in enriched taralli, it remained unchanged up to the end of the storage period (0.018 in *taralli* OPL and 0.024 µg/g DW in *taralli* OPC). 

## 3. Materials and Methods 

### 3.1. Raw Material

Semolina from durum wheat (cv Senatore Cappelli) was used to prepare *taralli*. Fermented OP samples from Cellina di Nardò and Leccino cultivars were produced following the method described by Tufariello et al. [13,16]. Briefly, OP obtained by a local olive mill (Murrone, Caprarica, Lecce, Italy), milled using a Multi-Phase Decanter technology (Leopard, Pieralisi Maip S.p.A., Jesi, AN, Italy), was subjected to heat treatment at 121 °C for 4 min. Then, OP samples were diluted 1:1 with distilled water and added with 0.5% (*w*/*v*) yeast extract, 0.5% (*w*/*v*) peptone, 0.5% (*w*/*v*) glucose in 10 kg capacity vessels. OP fermentations were carried out for 50 days at ambient temperature (18–22 °C) by the sequential inoculum of the yeast *Saccharomyces cerevisiae* ISPA-LE-KI 30-1 and then (after 30 days) with *Leuconostoc mesenteroides* ISPA-LE-BT3-35. Fermented OP samples were heat-treated at 90 °C for 2 min.

### 3.2. Chemicals

High purity standards for the qualitative-quantitative determination of fatty acids (palmitic, palmitoleic, heptadecanoic, stearic, oleic, linoleic, linolenic, arachidic, eicosenoic, behenic), α-T, triterpenic acids (maslinic and oleanoic acids), hexanal, and 2-methylpropyl acetate, as well as all High-Performance Liquid Chromatography (HPLC) grade solvents, were all purchased from Sigma–Aldrich (Milan, Italy). Tyrosol, hydroxytyrosol, and verbascoside were purchased from Fluka (Milan, Italy), Cabru s.a.s. (Arcore, Milan, Italy), and Extrasynthese (Genay Cedex, France), respectively. Oleacein and oleocanthal were obtained from PhytoLab GmbH & Co. (Vestenbergsgreuth, Germany). Carotenoids (lutein and β-carotene) and β-T3 standards were purchased from CaroteNature (Lupsingen, Liestal, Switzerland) and Cayman chemicals (Ann Arbor, MI, USA), respectively. 

### 3.3. Sample Preparation

*Taralli* were prepared by mixing wheat flour (1 kg), water (0.4 L), salt (0.02 kg), extra virgin olive oil (100 g), and or fermented OP (200 g) with a diving arm kneader for 20 min. Then, the dough was left to rest for 45 min, and after this time, was sheeted in taralli shape, round with a hole in the middle, of about 3 cm in diameter and 8 mm in thickness. *Taralli* samples were packed in 500 g transparent bags and stored in the real marketplace storage conditions (temperature 25 °C and light exposure, 12 h/day, to an intensity of 500 lux have been electronically controlled), during 180 days with samples analyses repeated every month.

### 3.4. Polyphenols Extraction and Analysis

Polyphenols were extracted, as reported by Servili et al. [53], with some modifications. Twenty grams of CTRL, OPC, and OPL *taralli* samples were ground with a blender (Osterizer Blender Cycle Blend Pulse 4153-50, Sunbeam Products, Inc., Boca Raton, FL, USA), and then 5 g was picked and extracted with 50 mL of methanol/water (80:20, *v*/*v*) containing 20 mg/L of butylated hydroxytoluene (BHT). The methanol was evaporated, and the aqueous extract was used for solid-phase extraction (SPE) of phenols. The SPE procedure was performed by loading 1 mL of the aqueous extract into a 1000 mg Bond Elut Jr-C18 cartridge (Agilent Technologies, Santa Clara, CA, USA), using 50 mL of methanol as the eluting solvent. The extract was evaporated under vacuum at 30 °C, the residue was dissolved in 1 mL of methanol and analyzed, as described by Servili et al. [53], using an 1100 Series HPLC system (Agilent Technologies, Santa Clara, CA, USA). For the quantitative analysis, an external calibration curve was constructed for each compound, except for isoverbascoside; the isoverbascoside was quantified using the response factor of verbascoside. 

### 3.5. Triterpenic Acids Extraction and Analysis

Triterpenic acids were extracted, as described by Durante et al. [10], from triplicate aliquots (1 g) of CTRL, OPC, and OPL *taralli* samples. Samples were extracted six times with 4 mL of methanol/ethanol (1:1, *v*/*v*) in a Labsonic LBS1-10 ultrasonic bath (Falc Instruments, Treviglio (Bg), Italy). Extracts were combined, evaporated to dryness, redissolved with 1 mL methanol, and analyzed, according to Durante et al. [10], using an 1100 Series HPLC system (Agilent Technologies, Santa Clara, CA, USA) equipped with a Luna column (5 μm, 250 × 4.6mm) (Phenomenex, Torrance, CA, USA). 

### 3.6. Tocochromanols and Carotenoids Extraction and Analysis

Tocochromanols (tocopherols and tocotrienols) and carotenoids were extracted from triplicate aliquots of CTRL, OPC, and OPL *taralli* samples, as reported by Padalino et al. [11]. The extracts were assayed, as described by Durante et al. [10], using an 1100 Series HPLC system (Agilent Technologies, Santa Clara, CA, USA) equipped with a reverse-phase C30 column (5 µm, 250 × 4.6 mm) (YMC Inc., Wilmington, NC, USA).

### 3.7. Total Lipid Extraction and Determination

Total lipids were extracted from triplicate aliquots (1 g) of CTRL, OPC, and OPL *taralli* samples with 5 mL of *n*-hexane and stirred with a mechanical stirrer (300 rpm) at 4 °C for 16 h. After centrifugation at 6000× *g* for 5 min, the organic phase was evaporated to dryness under a stream of nitrogen. Lipids extracts were subjected to fatty acid derivatization, as described by Durante et al. [8]. Briefly, a methanolic solution (3 mL) of 0.5 M KOH was added to the samples, after incubation at 100 °C for 5 min and cooling. Boron trifluoride in methanol (2 mL, 12% *w*/*v*) was added, and samples were incubated at 100 °C for 30 min. To the reaction mixture, 1 mL of NaCl (0.6% *w*/*v*) and 1 mL of *n*-hexane were added. The organic upper phase was analyzed by GC-MS in accordance to Durante et al. [8] using an Agilent 5977E Series GC/MS system (Agilent Technologies, Santa Clara, CA, USA) equipped with a DB-WAX column (60 m, 0.25 mm i.d., 0.25 mm film thickness) (Agilent Technologies, Santa Clara, CA, USA).

### 3.8. Headspace Solid-Phase Microextraction (SPME) and GC/MS Analysis of Hexanal

Triplicate aliquots (0.3 g) of CTRL, OPC, and OPL *taralli* samples with 3 mL saturated aqueous solution of NaCl were placed in a 20 mL vial tightly capped with polytetrafluoroethylene (PTFE) septum. The 2-Methylpropyl acetate as an internal standard at a concentration of 3 µg/g was added, and the mixture was homogenized for 2 min using a laboratory vortex shaker. Hexanal was sampled by SPME. The sample was maintained at 40 °C for 20 min, and after that, the fiber was exposed to the headspace for 30 min at 40 °C. The analyses of hexanal were conducted with an Agilent Technologies 7890B GC, equipped with a “Multimode Injector” (MMI) 7693A injector (Agilent Technologies, Santa Clara, CA, USA) and a thermostated PAL3 RSI 120 autosampler equipped with a fiber conditioning module and agitator (CTC Analytics AG, Zwingen, Switzerland), and the detection system was an Agilent 5977B single quadrupole GC/MSD with EI Extractor (XTR) source (Agilent Technologies, Santa Clara, CA, USA). For the analysis of hexanal, the fiber was thermally desorbed in the hot GC injector port for 5 min at 250 °C with the injector set in splitless mode. The volatile compounds were separated with a DB-WAXetr column (50 m, 0.32 mm i.d., 1 μm film thickness) (Agilent Technologies, Santa Clara, CA, USA) using helium as carrier gas at a constant flow rate of 1.7 mL/min. The GC oven heating program started at 35 °C with this temperature held for 4 min, then increased to 150 °C at a rate of 4 °C/min, increased to 180 °C at a rate of 8 °C/min held for 2 min, and finally increased to 210 °C at a rate of 11 °C/min, this temperature was held for 13.77 min. The temperature of the transfer line was fixed at 215 °C. The MSD was operated in the electron ionization (EI) mode, at ionization energy of 70 eV, in scan mode, with scanning in the mass range of *m*/*z* 25–350 a.m.u. at a scan rate of 4.3 scan/s and the MS source and the MS quad temperatures of 190 °C and 150 °C, respectively. The hexanal was identified by comparison of their mass spectrum and retention time with that of the authentic reference compound. This volatile compound was quantified by standardizing the peak area of hexanal with respect to the peak area of the internal standard (2-methylpropyl acetate), according to Lu Xiao et al. [54].

### 3.9. Statistical Analysis

The data are the mean values of replicate measurements (*n* = 3) with standard deviation. Student’s t-test was applied to the chemical characterization of CTRL *taralli* and enriched *taralli* with fermented olive paste from Cellina di Nardò (OPC) or Leccino (OPL) olive cultivars. A one-way analysis of variance (ANOVA) and the Tukey HSD post hoc test were applied to the time course of fatty acids, polyphenols, triterpenic acids, tocochromanols, and carotenoids. All statistical comparisons were performed using SigmaStat version 11.0 software (Systat Software Inc., Chicago, IL, USA).

## 4. Conclusions

In this study, the fermented olive paste by-product obtained from black olives (cv Cellina di Nardò and Leccino), due to its richness in bioactive compounds, was exploited to enhance nutritional and health-promoting properties of *taralli*, a typical Italian bakery product. Our data showed that *taralli* enriched with 20% of fermented OP were a convenient source of polyphenols, triterpenic acids, tocochromanols, and carotenoids and that the main components of this new types of *taralli* were hydroxytyrosol, tyrosol, verbascoside, oleacin, oleocanthal, maslinic acid, α-T, and lutein. During storage in the usual conditions of retailer shelves (25 °C and 500 lux), the enriched *taralli* maintained a low amount of saturated fatty acids and high levels of polyphenols, triterpenic acids, tocochromanols, and carotenoids up to about 90 days of storage. On the other hand, after 180 days of storage, we observed a drastic reduction in the total polyphenols (>60%), triterpenic acids (>67%), tocochromanols (>89%), and carotenoids (>74%). The obtained data highlighted, moreover, that fermented OP addition significantly reduced the level of hexanal compared to CTRL *taralli*. The results reported in this study encourage further studies to extend shelf-life and maintain the nutritional value of enriched *taralli* possibly using modified atmosphere packaging technique, in view of fulfilling consumer trend for healthy products. Furthermore, studies are required to assess if the utilization of *taralli* enriched with fermented OP would enhance human health performance and prevention from certain diseases.

This rather pioneering study is an example of how the circular economy could be applied to depleting food resources. Indeed, the use of microbial fermentation, a well-known strategy for food preservation and nutritional enhancement, is useful to convert a currently considered vegetable waste into functional ingredients and to produce enriched foods for the human diet.

## Figures and Tables

**Table 1:** Chemical characterization of control (CTRL) and *taralli* enriched with fermented olive paste from Cellina di Nardò (OPC) or Leccino (OPL) olive cultivars.

	*Taralli*		
	CTRL	OPC	OPL
**Fatty acids (relative percentage)**			
Palmitic acid (C16:0)	15.41 ± 1.28 ^a^	15.32 ± 1.50 ^a^	15.29 ± 0.98 ^a^
Palmitoleic acid (C16:1)	1.60 *±* 0.04 ^a^	1.58 ± 0.03 ^a^	1.61 ± 0.04 ^a^
Heptadecanoic acid (C17:0)	<0.01	<0.01	<0.11
Stearic acid (C18:0)	1.99 *±* 0.43 ^a^	2.03 ± 0.51 ^a^	2.04 ± 0.44 ^a^
Oleic acid (C18:1 n-9)	66.66 *±* 2.91 ^a^	66.04 ± 2.81 ^a^	66.54 ± 2.59 ^a^
Linoleic acid (C18:2n-6)	12.46 ± 0.85 ^a^	13.16 ± 1.05 ^a^	12.61 ± 0.35 ^a^
Linolenic acid(C18:3 n-3)	0.96 ± 0.05 ^a^	0.98 ± 0.04 ^a^	0.97 ± 0.05 ^a^
Arachidic acid (C20:0)	0.36 *±* 0.04 ^a^	0.37 ± 0.05 ^a^	0.37 ± 0.04 ^a^
*cis*-Eicosenoic acid (C20:1c)	0.33 *±* 0.05 ^a^	0.34 ± 0.06 ^a^	0.33 ± 0.07 ^a^
Behenic acid (C22:0)	0.23 *±* 0.05 ^a^	0.18 ± 0.04 ^a^	0.13 ± 0.04 ^a^
SFA	17.99 ± 1.80 ^a^	17.90 ± 2.10 ^a^	17.94 ± 1.50 ^a^
MUFA	68.59 ± 3.00 ^a^	67.96 ± 2.90 ^a^	68.48 ± 2.70 ^a^
PUFA	13.42 ± 0.90 ^a^	14.14 ± 1.09 ^a^	13.58 ± 0.40 ^a^
**Polyphenols (µg/g DW)**			
Hydroxytyrosol (3,4-DHPEA)	nd	905 ± 2	824 ± 1
Tyrosol (*p*-HPEA)	nd	81 ± 2	53 ± 3
Verbascoside	nd	202 ± 2	70 ± 3
Isoverbascoside *	nd	nd	36 ± 1
Oleacin (3,4-DHPEA-EDA)	nd	146 ± 6	33 ± 3
Oleocanthal (*p*-HPEA-EDA)	nd	43 ± 1	17 ± 1
Total	nd	1377 ± 13	1016 ± 12
**Triterpenic acids (µg/g DW)**			
Maslinic acid	nd	63.74 ± 3.01	68.93 ± 5.53
Oleanoic acid	nd	16.52 ± 1.12	16.99 ± 1.87
*Total*	nd	*80.26 ± 4.13*	*80.92 ± 7.40*
**Tocochromanols (µg/g DW)**			
α-T	10.31 ± 1.06 ^b^	21.26 ± 1.63 ^a^	15.63 ± 1.59 ^a^
β-T3	3.75 ± 0.45 ^b^	9.01 ± 0.03 ^a^	6.59 ± 0.96 ^a^
Total	14.06 ± 1.51 ^b^	30.27 ± 1.66 ^a^	22.22 ± 2.55 ^a^
**Carotenoids (µg/g DW)**			
Lutein	0.77 ± 0.06 ^b^	1.04 ± 0.03 ^a^	0.93 ± 0.03 ^a^
β carotene	0.06 ± 0.001 ^b^	0.13 ± 0.01 ^a^	0.10 ± 0.01 ^a^
Total	0.83 ± 0.06 ^b^	1.17 ± 0.04 ^a^	1.03 ± 0.14 ^b^

Significance: nd, not-detected; * tentative of identification. Data represent the mean ± standard deviation of three replicate measurements (*n* = 3). DW, dried weight; SFA, saturated fatty acid; MUFA, monosaturated fatty acids; PUFA, polyunsaturated fatty acid; Hydroxytyrosol (3,4-DHPEA), 3,4-(dihydroxyphenyl)ethanol; Tyrosol (*p*-HPEA), *p*-(hydroxyphenyl)ethanol; Oleacin (3,4-DHPEA-EDA), dialdehydic form of decarboxymethyl elenolic acid linked to (3,4-dihydroxyphenyl) ethanol; Oleocanthal (*p*-HPEA-EDA), dialdehydic form of decarboxymethyl elenolic acid linked to (*p*-hydroxyphenyl)ethanol; α-T, α-tocopherol; β-T3, β-tocotrienol. Different letters indicate significant differences (Student’s test, *p* < 0.05) within the same row (CTRL *taralli* vs. enriched *taralli*).

**Table 2:** Changes in fatty acid composition during the storage of CTRL *taralli* and *taralli* enriched with fermented olive paste from Cellina di Nardò (OPC) or Leccino (OPL) olive cultivars at 25 °C, 500 lux, and in clear plastic bag for 180 days.

Time Storage (days)	*Taralli*											
	CTRL				OPC				OPL			
	SFA	MUFA	PUFA	PUFA/SFA	SFA	MUFA	PUFA	*PUFA/SFA*	SFA	MUFA	PUFA	PUFA/SFA
	%				%				%			
0	19.99 ± 1.80 ^b^	68.59 ± 3.01 ^a^	13.42 ± 0.90 ^a^	0.67	17.90 ± 2.10 ^a^	67.96 ± 2.90 ^a^	14.14 ± 1.09 ^a^	*0.79*	17.94 ± 1.50 ^b^	68.48 ± 2.70 ^a^	13.58 ± 0.40 ^a^	0.76
30	19.01 ± 1.8 ^b^	67.75 ± 3.10 ^a^	13.25 ± 1.10 ^ab^	0.69	18.43 ± 1.99 ^a^	68.14 ± 2.90 ^a^	13.43 ± 1.09 ^a^	*0.73*	17.85 ± 1.90 ^b^	67.87 ± 2.20 ^a^	14.28 ± 0.99 ^a^	0.80
60	19.94 ± 1.87 ^b^	67.57 ± 4.10^a^	11.08 ± 0.99 ^b^	0.56	19.78 ± 1.77 ^a^	67.01 ± 3.21 ^a^	13.22 ± 1.01 ^a^	*0.67*	18.01 ± 1.68 ^b^	67.90 ± 3.10 ^a^	14.10 ± 1.10 ^a^	0.78
90	21.13 ± 2.10 ^a^	67.87 ± 3.80^a^	11.01 ± 0.98 ^b^	0.52	20.51 ± 2.10 ^a^	67.49 ± 2.90 ^a^	12.01 ± 0.87 ^a^	*0.58*	19.21 ± 2.20 ^b^	67.85 ± 3.10 ^a^	12.94 ± 0.76 ^ab^	0.67
120	21.58 ± 1.80 ^a^	67.57 ± 3.70 ^a^	10.85 ± 0.88 ^b^	0.50	22.08 ± 2.00 ^a^	67.47 ± 3.21 ^a^	10.45 ± 0.79 ^b^	*0.47*	19.88 ± 2.10 ^a^	67.81 ± 3.30 ^a^	12.31 ± 0.65 ^ab^	0.62
150	22.51 ± 0.90 ^a^	67.49 ± 4.1 ^a^	10.01 ± 0.08 ^b^	0.44	22.50 ± 1.80 ^a^	67.50 ± 4.52 ^a^	10.01 ± 0.05 ^b^	*0.44*	21.54 ± 1.20 ^a^	67.40 ± 5.60 ^a^	11.06 ± 0.90 ^bc^	0.51
180	26.01 ± 2.01 ^a^	67.50 ± 4.50 ^a^	6.52 ± 0.10 ^b^	0.25	23.01 ± 1.90 ^a^	68.01 ± 4.69 ^a^	9.03 ± 0.21 ^b^	*0.39*	23.08 ± 1.70 ^a^	67.00 ± 4.71 ^a^	9.92 ± 0.80 ^c^	0.43

Data are the mean ± standard deviation of three independent replicates (*n* = 3). Data were submitted to one-way analysis of variance (ANOVA), Tukey’s test was applied to compare the experimental times (0, 30, 60, 90, 120, 150, 180 days) within the same sample for each classification of fatty acids (SFA, MUFA, and PUFA). Different letters mean significant difference at *p* < 0.05.

## Supplementary Materials

The following are available online, Figure S1: HPLC chromatogram of polyphenol extracts from *Taralli* CTRL, OPC, and OPL. Samples were recorded with DAD at 278 nm. Peak numbers: 1, Hydroxytyrosol; 2, Tyrosol; 3, Verbascoside; 4, Oleacin; 5, Isoverbascoside; 6, Oleocanthal, Table S1: Polyphenols composition in virgin olive oil from Cellina di Nardò and Leccino olive cultivars.
