# Supplementary material for: Bioactive Compounds and Stability of a Typical Italian Bakery Products “Taralli” Enriched with Fermented Olive Paste
Source: Molecules. 2019 Sep 6;24(18):3258. doi: 10.3390/molecules24183258 (PMC6766877; doi:10.3390/molecules24183258)
Supplement: Supplementary file 1 [file molecules-24-03258-s001.pdf]

Figure S1. HPLC chromatogram of polyphenol extracts from Taralli CTRL, OPC and OPL. Samples were recorded with DAD at 278 nm. Peak numbers: 1, Hydroxytyrosol; 2, Tyrosol; 3, Verbascoside; 4, Oleacin; 5, Isoverbascoside; 6, Oleocanthal.

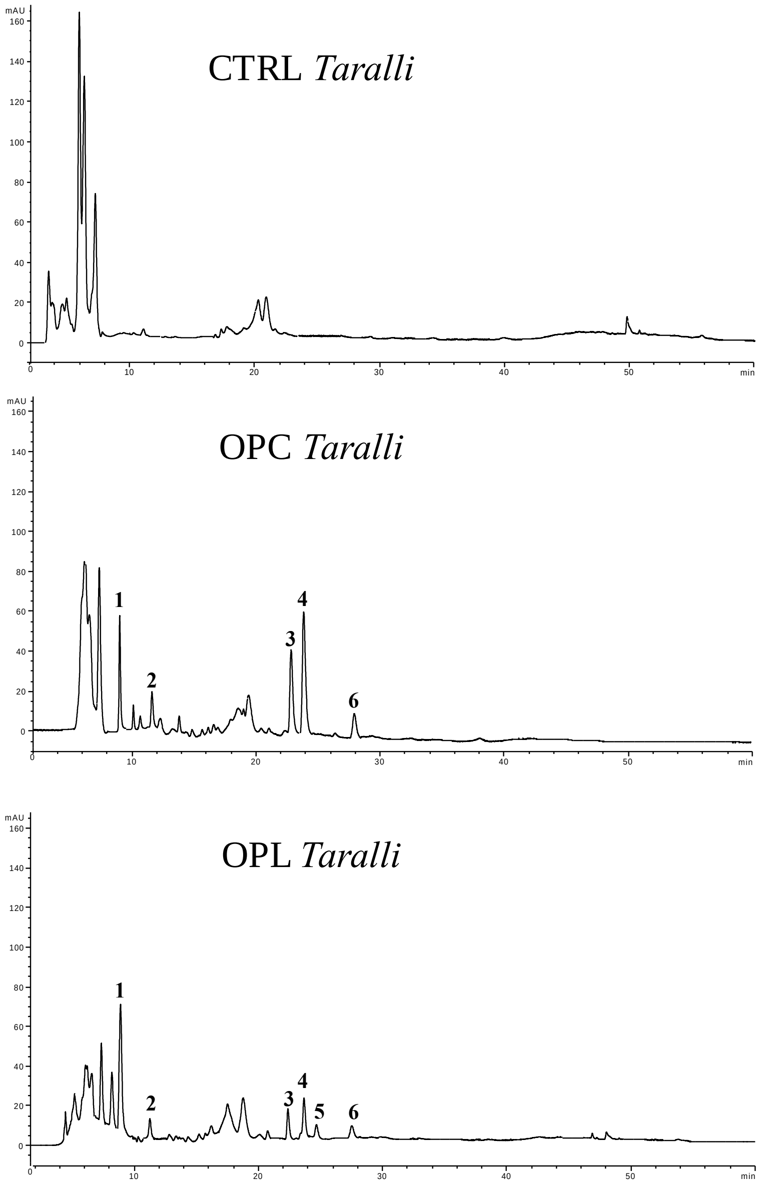

Table S1. Polyphenols composition of virgin olive oil from Cellina di Nardò and Leccino olive cultivars.

	Cellina di Nardò	Leccino
	mg/100g	
Hydroxytyrosol (3,4-DHPEA)	0.26±0.08	0.42±0.001
Tyrosol (p-HPEA)	1.38±0.05	0.38±0.03
Oleuropein	47.08±2.44	9.52±0.86
Quercetin	4.74±0.14	3.05±0.29
Pinoresinol	nd	0.11±0.001
Total	*53.46±2.71*	*13.48±1.18*

Significance: nd, not detected. Data represent the mean ± standard deviation of three replicate measurements (n = 3).
